# Comparison of two Mn^IV^Mn^IV^-bis-μ-oxo complexes {[Mn^IV^(N_4_(6-Me-DPEN))]_2_(μ-O)_2_}^2+^ and {[Mn^IV^(N_4_(6-Me-DPPN))]_2_(μ-O)_2_}^2+^

**DOI:** 10.1107/S2056989020004557

**Published:** 2020-06-09

**Authors:** Michael K. Coggins, Alexandra N. Downing, Werner Kaminsky, Julie A. Kovacs

**Affiliations:** a The Department of Chemistry, University of Washington, Box 351700, Seattle, Washington 98195-1700, USA

**Keywords:** manganese, high-valent, metal-oxo, transition metal, crystal structure

## Abstract

The addition of *tert*-butyl hydro­peroxide (^*t*^BuOOH) to two structurally related Mn^II^ complexes containing *N,N*-bis­(6-methyl-2-pyridyl­meth­yl)ethane-1,2-di­amine (6-Me-DPEN) and *N,N*-bis­(6-methyl-2-pyridyl­meth­yl)propane-1,2-di­amine (6-Me-DPPN) results in the formation of high-valent bis-oxo complexes, namely di-μ-oxido-bis­{[*N*,*N*-bis­(6-methyl-2-pyridylmeth­yl)ethane-1,2-di­amine]­manganese(II)}(*Mn*—*Mn*) bis­(tetra­phenyl­borate) dihydrate, [Mn(C_16_H_22_N_4_)_2_O_2_](C_24_H_20_B)_2_·2H_2_O or {[Mn^IV^(N_4_(6-Me-DPEN))]_2_(*μ*-O)_2_}(2BPh_4_)(2H_2_O) (**1**) and di-μ-oxido-bis­{[*N*,*N*-bis­(6-methyl-2-pyridylmeth­yl)propane-1,3-di­amine]­manganese(II)}(*Mn*—*Mn*) bis­(tetra­phenyl­borate) diethyl ether disolvate, [Mn(C_17_H_24_N_4_)_2_O_2_](C_24_H_20_B)_2_·2C_4_H_10_O or {[Mn^IV^(N_4_(6-MeDPPN))]_2_(*μ*-O)_2_}(2BPh_4_)(2Et_2_O) (**2**). Complexes **1** and **2** both contain the ‘diamond core’ motif found previously in a number of iron, copper, and manganese high-valent bis-oxo compounds. The flexibility in the propyl linker in the ligand scaffold of **2**, as compared to that of the ethyl linker in **1**, results in more elongated Mn—N bonds, as one would expect. The Mn—Mn distances and Mn—O bond lengths support an Mn^IV^ oxidation state assignment for the Mn ions in both **1** and **2**. The angles around the Mn centers are consistent with the local pseudo-octa­hedral geometry.

## Abstract 2

The addition of *tert*-butyl hydro­peroxide (^*t*^BuOOH) to two Mn^II^ complexes, differing by a small synthetic alteration from an ethyl to a propyl linker in the ligand scaffold, results in the formation of the high-valent bis-oxo complexes {[Mn^IV^(N_4_(6-Me-DPEN))]_2_(μ-O)_2_}^2+^ (**1**) and {[Mn^IV^(N_4_(6-Me-DPPN))]_2_(μ-O)_2_}^2+^ (**2**).

## Chemical context   

A heterometallic cubane cluster, Mn_dang_CaMn_3_O_5_, referred to as the oxygen-evolving complex (OEC), is involved in photosynthetic catalytic water oxidation (Umena *et al.*, 2011 ▸). The cluster is housed in the enzyme photosystem II (PSII) and consists of high-valent Mn^III/IV^ ions linked by oxo bridges and one dangling Mn^IV/V^ ion. Water oxidation is thermodynamically unfavorable, and requires an energy input of 359 kJ mol^−1^ that is provided by sunlight (Yano & Yachandra, 2014 ▸). Although the exact details of the mechanism for water oxidation are unknown, two water mol­ecules are thought to bind to the cluster to produce one equivalent of di­oxy­gen, four electrons, and four protons (Kok *et al.*, 1970 ▸). Sequential oxidation of the cluster, starting with the Ca^II^Mn^IV^Mn_3_^III^O_5_ core, generates partially oxidized states, *S_i_* (where *i* = number of stored oxidizing equivalents), which store oxidizing equivalents in preparation for O—O bond formation and O_2_ release (Hatakeyama *et al.*, 2016 ▸; Lohmiller *et al.*, 2017 ▸; Renger, 2011 ▸; Yano & Yachandra, 2014 ▸). Very little is known about the key OEC-catalyzed O—O bond-forming step, because it occurs following the rate-determining step (Retegan *et al.*, 2016 ▸). Proposed mechanisms for O—O bond formation involve either nucleophilic attack by an *M*—OH group (*M* = Mn or Ca) at an electrophilic Mn^V^≡O site, or radical coupling between two Mn^IV^ oxyl radicals to afford an unobserved peroxo inter­mediate (Hatakeyama *et al.*, 2016 ▸; Lohmiller *et al.*, 2017 ▸; Renger, 2011 ▸; Yano & Yachandra, 2014 ▸). Developing a wide base of chemical information on a variety of Mn—O species similar to the fragments implicated in the key O—O bond-forming step should aid the development of a detailed understanding of photosynthetic water oxidation. Fundamental concepts obtained from these studies can then be applied towards the maintenance of stable energy reserves and improve the world’s energy economy by storing solar energy in chemical bonds (Lewis, 2016 ▸).
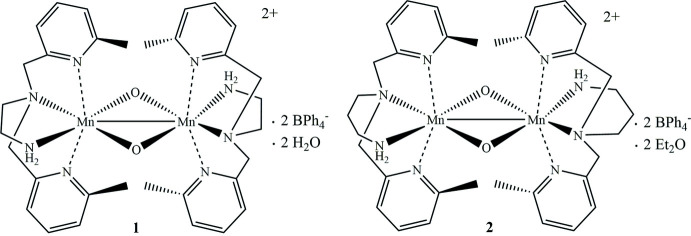


**Figure 1:**
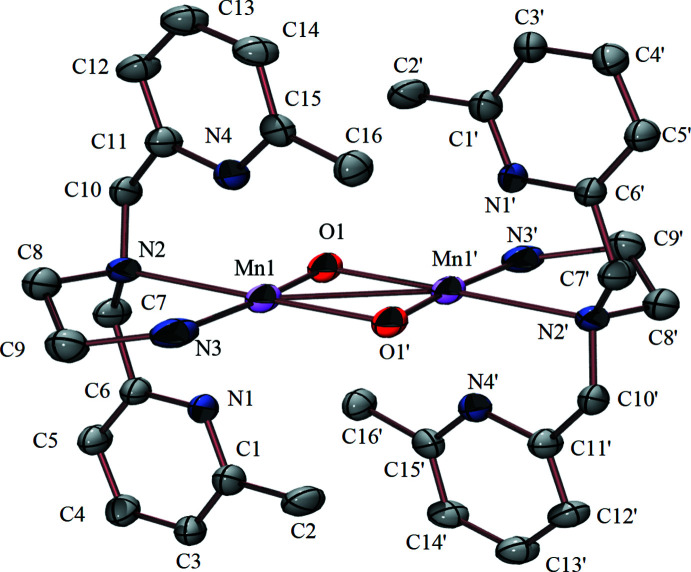
Ellipsoid plot of {[Mn^IV^(N_4_(6-Me-DPEN))]_2_(*μ*-O)_2_}^2+^ (**1**) showing the atom-labeling scheme. The anions and all hydrogen atoms have been removed for clarity. Displacement ellipsoids are drawn at the 50% probability level. Symmetry code for primed atoms *-x*, −*y* + 1, −*z* + 1.

A key step in OEC-catalyzed water oxidation involves the formation of a peroxo O—O bond prior to di­oxy­gen evolution. Previous work by the Kovacs group has facilitated an understanding of the metal-ion properties that favor peroxo O—O bond formation *versus* cleavage, and O_2_ binding *versus* release (Coggins *et al.*, 2012 ▸, 2013**a* ▸,*b* ▸,c* ▸; Coggins & Kovacs, 2011 ▸; Poon *et al.*, 2019 ▸). Reversible di­oxy­gen binding and release was shown to strongly correlate with metal-ion Lewis acidity. Superoxo, peroxo, and reactive mixed-valent Mn^III^Mn^IV^ bis-oxo inter­mediates were shown to form. In addition, thiol­ate ligands were shown to increase the HAT (hydrogen-atom transfer) reactivity of putative Mn^IV^Mn^IV^ dimer inter­mediates, precluding their isolation (Poon *et al.*, 2019 ▸). In contrast, alkoxide derivatives [Mn^III^(O^Me2^N_4_(6-Me-DPEN))](BPh_4_) (**3**) and [Mn^III^(O^Me2^N_4_(6-Me-DPPN))](BPh_4_)⋯Et_2_O (**4**) (Coggins *et al.*, 2020 ▸) react with ^*t*^BuOOH to form ultimately the high-valent complexes described herein: {[Mn^IV^(N_4_(6-Me-DPEN))]_2_(μ-O)_2_}^2+^ (**1**) and {[Mn^IV^(N_4_(6-Me-DPPN))]_2_(μ-O)_2_}^2+^ (**2**). The isolation and crystallographic characterization of the bis-oxo complexes **1** and **2** (Figs. 1 ▸ and 2 ▸, formed *via* alkyl­peroxo Mn—OO^*t*^Bu inter­mediates (Coggins *et al.*, 2020 ▸), further expands the available library of high-valent Mn–oxo dimers (Mullins & Pecoraro, 2008 ▸), demonstrating the stability of the metal–oxo diamond core described previously (Que & Tolman, 2002 ▸).

## Structural Commentary   

### Complex 1   

Complex **1** possesses a non-crystallographic *C*_2_ rotation axis and the two Mn centers are crystallographically equivalent across an inversion center (−*x*, 1 − *y*, 1 − *z*). The Mn ion of **1** is in a pseudo-octa­hedral environment, with small deviations in the O—Mn—N angles relative to an ideal octa­hedral geometry: O1—Mn1—N1 = 93.76 (12), O1—Mn1—N2 = 92.13 (12), O1—Mn1—N3 = 174.90 (12), and O1—Mn1—N4 = 95.77 (12)°. As is true for all diamond cores, the O1—Mn1—O1′ angle is slightly compressed at 85.53 (12)°. Metrical parameters, Mn1—O1 = 1.829 (3) Å and Mn1—O1′= 1.835 (3) Å (Table 1 ▸) fall within the reported range (1.8 to 1.9 Å) for oxo-bridged Mn^IV^ complexes (Krewald *et al.*, 2013 ▸; Mullins & Pecoraro, 2008 ▸; Torayama *et al.*, 1998 ▸). The pyridine nitro­gen atoms are outside the typical bonding range, but are oriented towards the Mn ion at distances of Mn1—N1= 2.348 (3) Å and Mn1—N4 = 2.368 (3) Å. Unfavorable steric inter­actions involving the methyl group at the 6-position of the pyridine arm are likely to be responsible for the longer Mn—N(1,4) distances. Manganese–nitro­gen distances involving the amine arms fall within the normal Mn—N range (1.9 to 2.1 Å) for Mn^IV^. The bond involving the tertiary amine [Mn1—N2 = 2.123 (3) Å] is slightly longer than that involving the secondary amine [Mn1—N3 = 2.111 (4) Å]. The Mn1⋯Mn1′ separation of 2.6899 (15) Å, falls within the normal range (2.6 to 2.8 Å) for bis-oxo-bridged Mn^IV^Mn^IV^ dimers containing a diamond core. Complex **1** crystallizes with two crystallographically equivalent tetra­phenyl­borate counter-ions and two crystallographically equivalent water mol­ecules. The water mol­ecule is disordered over two sites with site occupancies refined to 0.870 (12) and 0.130 (12) for O2 and O2*B* respectively, with the applied constraint that both together give 100% occupancy.

### Complex 2   

Complex **2** also sits on an inversion center (1 − *x*, 2 − *y*, 1 − *z*), making the two Mn atoms crystallographically equivalent. There is disorder in the position of the propyl linker carbon atoms (C1, C2, C3). The site occupancies of N3, C1–C3 and N3*B*, C1*B*–C3*B* refined to 0.804 (5) and 0.196 (5), respectively, with the constraint of both together giving 100% occupancy. The Mn ion of **2** is again in a pseudo-octa­hedral environment, with small deviations in O—Mn—N angles relative to ideal octa­hedral geometry: O1—Mn1—N1 = 106.39 (7), O1—Mn1—N2 = 174.90 (7), O1—Mn1—N3 = 89.11 (13), and O1—Mn1—N4 = 103.70 (6)°. Again, as is true for all diamond cores, the O1—Mn1—O1′ angle of **2** is slightly compressed at 85.98 (7)°, and is similar to that in **1**. Metrical parameters, Mn—O1 = 1.8325 (15) and Mn—O1′ = 1.8349 (15) Å, are also similar to those found in **1**, and fall within the reported range (1.8 to 1.9 Å) for oxo-bridged Mn^IV^ complexes. The pyridine nitro­gen atoms are once again further from the Mn ions than expected for a formal Mn—N bond, but are oriented towards Mn at distances of Mn1—N1 = 2.3251 (18) Å and Mn1—N4 = 2.3522 (18) Å. This bond elongation is likely to be due to steric inter­ference from the methyl groups at the 6-position of the pyridine rings. The nitro­gens on the amine arms are much closer to the Mn center, and fall within the normal Mn—N range (1.9 to 2.1 Å) for Mn^IV^. The Mn—N distance involving the tertiary amine [Mn1—N2 = 2.1828 (18) Å] is noticeably longer than that involving the secondary amine [Mn1—N3= 2.133 (6) Å]. The large difference between these bond lengths in **2**, relative to those of **1**, likely reflects the increased flexibility of the propyl linker in **2**. The Mn1—Mn1′ distance [2.6825 (7) Å] in **2** is essentially the same as that found in **1**, and falls within the normal range (2.6 to 2.8 Å) for bis-oxo-bridged Mn^IV^Mn^IV^ dimers containing a diamond core. Complex **2** crystallizes with two tetra­phenyl­borate counter-ions and two diethyl ether mol­ecules per cation.

## Database survey   

The structures of **1** and **2** are analogous to other reported Mn^IV^Mn^IV^(*μ*-O)_2_ dimers. The Mn1—Mn1′ distances of 2.6899 (15) Å in **1** and 2.6825 (7) Å in **2** are comparable to other literature examples (Krewald *et al.*, 2013 ▸; Mullins & Pecoraro, 2008 ▸; Torayama, *et al.*, 1998 ▸). The Mn—O bond lengths of 1.829 (3) and 1.835 (2) Å for **1** and 1.8350 (15) and 1.8325 (15) Å for **2** are also similar to literature reported values for Mn^IV^Mn^IV^(*μ*-O)_2_ dimers (Krewald *et al.*, 2013 ▸; Mullins & Pecoraro, 2008 ▸; Torayama *et al.*, 1998 ▸). The octa­hedral geometry of the Mn centers of both structures are very similar in terms of bond angles, all of which are close to the ideal 90 and 180°. The similarities in bond lengths and angles show that **1** and **2** contain a metal–oxo diamond core motif, previously observed in manganese, iron and copper complexes (Que & Tolman, 2002 ▸).

## Synthesis and crystallization   

### General methods   

All syntheses were performed using Schlenk-line tech­niques or under an N_2_ atmosphere in a glovebox. Reagents and solvents were purchased from commercial vendors, were of highest available purity and were used without further purification unless otherwise noted. MeOH (Na), MeCN (CaH_2_), and CH_2_Cl_2_ (CaH_2_) were dried and distilled prior to use. Et_2_O was rigorously degassed and purified using solvent purification columns housed in a custom stainless steel cabinet and dispensed by a stainless steel Schlenk-line (GlassContour). Complexes **3** and **4** were synthesized as described by Coggins *et al.* (2020 ▸).

### Synthesis of 1 and 2   

The addition of 1.5 equivalents of ^*t*^BuOOH to CH_2_Cl_2_ solutions of alkoxide-ligated **3** and **4** in an anaerobic cell at room temperature results in the formation of **1** and **2**, respectively. Single crystals of the isolated compounds in the form of brown plates for **1** and purple plates for **2** were obtained in up to 40% yield *via* slow evaporation and crystallization from CH_2_Cl_2_. Both reactions result in the loss of the Schiff-base arm present in the starting Mn^II^ complexes **3** and **4**, most probably because the reactions were performed in moist air (Coggins *et al.*, 2020 ▸).

## Refinement   

Crystal data, data collection and structure refinement details are summarized in Table 2 ▸. Scattering factors are taken from Waasmaier & Kirfel (1995 ▸). Hydrogen atoms were placed in geometrically idealized positions and constrained to ride on their parent atoms with C—H distances in the range 0.95–1.00 Å. Isotropic displacement parameters *U*_eq_ were fixed at 1.2*U*_eq_(C) or 1.5*U*_eq_(C-meth­yl). For the disordered water mol­ecule in complex **1**, the water was set-up as a rigid group free to rotate and move during refinement, with DFIX restraints between O and H and between both H per water. The displacement parameters of O2 and O2*B* were made the same with the EADP constraint. Hydrogen-atom isotropic displacement parameters were fixed at 1.5 times that of the water oxygen atoms. For the disorder in complex **2**, the geometry of both groups was set to be similar with the ‘SAME’ option. Displacement parameters of N3-N3*B*, C1-C1*B*, C2-C2*B*, and C3-C3*B* were restrained with the SIMU command at 0.005 strength.

## Supplementary Material

Crystal structure: contains datablock(s) global, Complex1, Complex2. DOI: 10.1107/S2056989020004557/cq2034sup1.cif

Structure factors: contains datablock(s) Complex1. DOI: 10.1107/S2056989020004557/cq2034Complex1sup4.hkl

Structure factors: contains datablock(s) Complex2. DOI: 10.1107/S2056989020004557/cq2034Complex2sup5.hkl

CCDC references: 1994292, 1994291

Additional supporting information:  crystallographic information; 3D view; checkCIF report

## Figures and Tables

**Figure 2:**
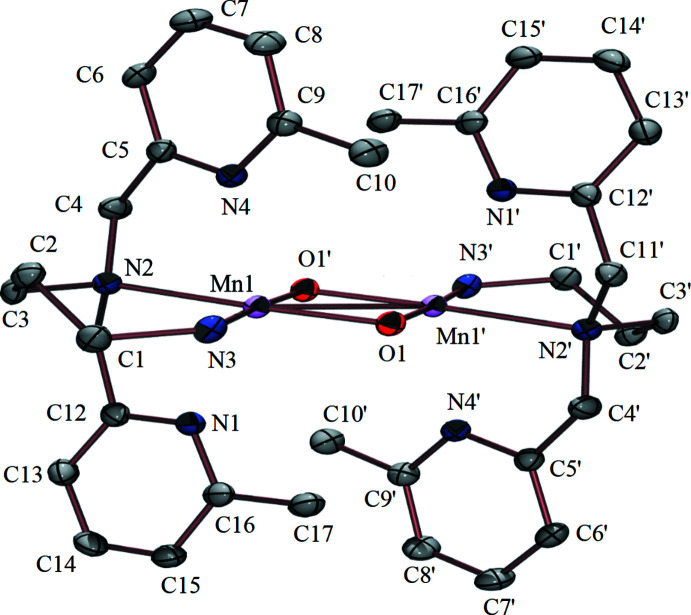
Ellipsoid plot of {[Mn^IV^(N_4_(6-Me-DPPN))]_2_(*μ*-O)_2_}^2+^ (**2**) showing the atom-labeling scheme. The anions, solvent, disorder, and hydrogen atoms have been removed for clarity. Displacement ellipsoids are drawn at the 50% probability level. Symmetry code for primed atoms: *-x +* 1, −*y* + 2, −*z* + 1.

**Table 1:** Comparison of key bond lengths and angles (Å, °) for complexes **1** and **2**

	Complex **1**	Complex **2**
Mn1—O1	1.829 (3)	1.8325 (15)
Mn1—O1′	1.835 (2)	1.8350 (15)
Mn1—N1	2.348 (3)	2.3251 (18)
Mn1—N2	2.123 (3)	2.1828 (18)
Mn1—N3	2.111 (4)	2.133 (6)
Mn1—N4	2.368 (3)	2.3522 (18)
Mn1—Mn1′	2.6899 (15)	2.6825 (7)
		
O1—Mn1—N1	93.76 (12)	106.39 (7)
O1—Mn1—N2	92.13 (12)	174.90 (7)
O1—Mn1—N3	174.90 (12)	89.11 (13)
O1—Mn1—N4	95.77 (12)	103.70 (6)
O1—Mn1—O1′	85.53 (3)	85.98 (7)

Symmetry codes for primed atoms are −*x*, 1 − *y*, 1 − *z* for **1** and 1 − *x*, 2 − *y*, 1 − *z* for **2**.

**Table 2:** Experimental details

	Complex **1**	Complex **2**
Crystal data		
Chemical formula	[Mn(C_16_H_22_N_4_)_2_O_2_](C_24_H_20_B)_2_·2H_2_O	[Mn(C_17_H_24_N_4_)_2_O_2_](C_24_H_20_B)_2_·2C_4_H_10_O
*M*_r_	1357.08	1497.34
Crystal system, space group	Triclinic, *P* 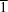	Monoclinic, *P*2_1_/*n*
Temperature (K)	100	100
*a*, *b*, *c* (Å)	12.169 (3), 12.404 (4), 13.845 (4)	15.9472 (16), 13.8380 (14), 17.5219 (17)
α, β, γ (°)	69.752 (7), 67.355 (8), 68.725 (7)	90, 91.123 (5), 90
*V* (Å^3^)	1744.7 (8)	3865.9 (7)
*Z*	1	2
Radiation type	Mo *K*α	Mo *K*α
μ (mm^−1^)	0.42	0.39
Crystal size (mm)	0.15 × 0.05 × 0.05	0.1 × 0.05 × 0.05

Data collection		
Diffractometer	Bruker APEXII CCD area-detector	Bruker APEXII CCD area-detector
Absorption correction	Multi-scan (*SADABS*; Bruker, 2007 ▸)	Multi-scan (*SADABS*; Bruker, 2007 ▸)
*T*_min_, *T*_max_	0.940, 0.979	0.915, 0.947
No. of measured, independent and observed [*I* > 2σ(*I*)] reflections	22505, 8374, 3541	138191, 9679, 7420
*R*_int_	0.099	0.068

Refinement		
*R*[*F* ^2^ > 2σ(*F* ^2^)], *wR*(*F* ^2^), *S*	0.073, 0.163, 0.97	0.049, 0.134, 1.05
No. of reflections	8374	9679
No. of parameters	439	517
No. of restraints	6	29
H-atom treatment	H-atom parameters constrained	H-atom parameters constrained
Δρ_max_, Δρ_min_ (e Å^−3^)	0.42, −0.46	0.66, −1.01

Computer programs: *APEX2* and *SAINT* (Bruker, 2007 ▸), *SHELXS97* and *SHELXL97* (Sheldrick, 2008 ▸), *SHELXL2014/7* (Sheldrick, 2015 ▸) and *ORTEP-3 for Windows* (Farrugia, 2012 ▸).

## supplementary crystallographic information

### Di-µ-oxido-bis{[N,N-bis(6-methyl-2-pyridilmethyl)ethane-1,2-diamine]manganese(II)}(Mn—Mn) bis(tetraphenylborate) dihydrate (Complex1) Crystal data

**Table:**

[Mn(C_16_H_22_N_4_)_2_O_2_](C_24_H_20_B)_2_·2H_2_O	*Z* = 1
*M**_r_* = 1357.08	*F*(000) = 716
Triclinic, *P*1	*D*_x_ = 1.292 Mg m^−^^3^
Hall symbol: -P 1	Mo *K*α radiation, λ = 0.71073 Å
*a* = 12.169 (3) Å	Cell parameters from 123 reflections
*b* = 12.404 (4) Å	θ = 3–20°
*c* = 13.845 (4) Å	µ = 0.42 mm^−^^1^
α = 69.752 (7)°	*T* = 100 K
β = 67.355 (8)°	Plate, brown
γ = 68.725 (7)°	0.15 × 0.05 × 0.05 mm
*V* = 1744.7 (8) Å^3^	

### Di-µ-oxido-bis{[N,N-bis(6-methyl-2-pyridilmethyl)ethane-1,2-diamine]manganese(II)}(Mn—Mn) bis(tetraphenylborate) dihydrate (Complex1) Data collection

**Table:**

Bruker APEXII CCD area-detector diffractometer	8374 independent reflections
Radiation source: fine-focus sealed tube	3541 reflections with *I* > 2σ(*I*)
Graphite monochromator	*R*_int_ = 0.099
φ and ω scans	θ_max_ = 28.5°, θ_min_ = 1.6°
Absorption correction: multi-scan (SADABS; Bruker, 2007)	*h* = −16→16
*T*_min_ = 0.940, *T*_max_ = 0.979	*k* = −16→16
22505 measured reflections	*l* = −18→18

### Di-µ-oxido-bis{[N,N-bis(6-methyl-2-pyridilmethyl)ethane-1,2-diamine]manganese(II)}(Mn—Mn) bis(tetraphenylborate) dihydrate (Complex1) Refinement

**Table:**

Refinement on *F*^2^	Primary atom site location: structure-invariant direct methods
Least-squares matrix: full	Secondary atom site location: difference Fourier map
*R*[*F*^2^ > 2σ(*F*^2^)] = 0.073	Hydrogen site location: mixed
*wR*(*F*^2^) = 0.163	H-atom parameters constrained
*S* = 0.97	*w* = 1/[σ^2^(*F*_o_^2^) + (0.0563*P*)^2^] where *P* = (*F*_o_^2^ + 2*F*_c_^2^)/3
8374 reflections	(Δ/σ)_max_ < 0.001
439 parameters	Δρ_max_ = 0.42 e Å^−^^3^
6 restraints	Δρ_min_ = −0.46 e Å^−^^3^

### Di-µ-oxido-bis{[N,N-bis(6-methyl-2-pyridilmethyl)ethane-1,2-diamine]manganese(II)}(Mn—Mn) bis(tetraphenylborate) dihydrate (Complex1) Special details

**Table:**

Experimental. 20 seconds exposure, 0.5 degree steps
Geometry. All esds (except the esd in the dihedral angle between two l.s. planes) are estimated using the full covariance matrix. The cell esds are taken into account individually in the estimation of esds in distances, angles and torsion angles; correlations between esds in cell parameters are only used when they are defined by crystal symmetry. An approximate (isotropic) treatment of cell esds is used for estimating esds involving l.s. planes.
Refinement. Refinement of F^2^ against ALL reflections. The weighted R-factor wR and goodness of fit S are based on F^2^, conventional R-factors R are based on F, with F set to zero for negative F^2^. The threshold expression of F^2^ > 2σ(F^2^) is used only for calculating R-factors(gt) etc. and is not relevant to the choice of reflections for refinement. R-factors based on F^2^ are statistically about twice as large as those based on F, and R- factors based on ALL data will be even larger.

### Di-µ-oxido-bis{[N,N-bis(6-methyl-2-pyridilmethyl)ethane-1,2-diamine]manganese(II)}(Mn—Mn) bis(tetraphenylborate) dihydrate (Complex1) Fractional atomic coordinates and isotropic or equivalent isotropic displacement parameters (Å^2^)

**Table:**

	*x*	*y*	*z*	*U*_iso_*/*U*_eq_	Occ. (<1)
C1	0.0240 (4)	0.1880 (4)	0.5092 (3)	0.0284 (10)	
C2	−0.1120 (3)	0.2320 (4)	0.5589 (3)	0.0329 (11)	
H2A	−0.1332	0.3172	0.5553	0.049*	
H2D	−0.1354	0.188	0.6344	0.049*	
H2C	−0.1567	0.2197	0.5196	0.049*	
C3	0.0849 (4)	0.0675 (4)	0.5299 (3)	0.0327 (11)	
H3	0.0406	0.0116	0.5799	0.039*	
C4	0.2083 (4)	0.0281 (4)	0.4788 (3)	0.0346 (11)	
H4	0.2497	−0.0544	0.4924	0.042*	
C5	0.2707 (4)	0.1112 (4)	0.4074 (3)	0.0327 (11)	
H5	0.3557	0.0861	0.3694	0.039*	
C6	0.2102 (4)	0.2291 (4)	0.3915 (3)	0.0285 (10)	
C7	0.2745 (3)	0.3250 (3)	0.3235 (3)	0.0309 (11)	
H7A	0.3089	0.3441	0.3678	0.037*	
H7B	0.3441	0.2953	0.2639	0.037*	
C8	0.1587 (4)	0.4141 (4)	0.1926 (3)	0.0348 (11)	
H8A	0.1694	0.3277	0.2061	0.042*	
H8B	0.2171	0.4391	0.1215	0.042*	
C9	0.0271 (4)	0.4810 (4)	0.1887 (3)	0.0362 (11)	
H9A	0.0215	0.5666	0.1544	0.043*	
H9B	0.0036	0.4487	0.1455	0.043*	
C10	0.2493 (3)	0.5339 (3)	0.2369 (3)	0.0320 (11)	
H10A	0.316	0.5237	0.169	0.038*	
H10B	0.2873	0.53	0.2901	0.038*	
C11	0.1599 (4)	0.6545 (3)	0.2166 (3)	0.0284 (10)	
C12	0.2000 (4)	0.7490 (4)	0.1419 (3)	0.0329 (11)	
H12	0.2838	0.739	0.0987	0.039*	
C13	0.1151 (4)	0.8603 (4)	0.1305 (3)	0.0373 (12)	
H13	0.1399	0.928	0.0798	0.045*	
C14	−0.0053 (4)	0.8701 (4)	0.1941 (3)	0.0367 (12)	
H14	−0.0641	0.9457	0.1883	0.044*	
C15	−0.0415 (4)	0.7715 (4)	0.2660 (3)	0.0325 (11)	
C16	−0.1737 (3)	0.7774 (4)	0.3317 (3)	0.0386 (12)	
H16A	−0.1767	0.7173	0.4002	0.058*	
H16B	−0.2154	0.7618	0.2918	0.058*	
H16C	−0.2151	0.8568	0.3458	0.058*	
C17	−0.3193 (4)	0.4439 (4)	0.1848 (3)	0.0298 (10)	
C18	−0.4283 (4)	0.5241 (4)	0.2286 (3)	0.0341 (11)	
H18	−0.4957	0.494	0.2792	0.041*	
C19	−0.4418 (5)	0.6461 (4)	0.2008 (4)	0.0457 (13)	
H19	−0.518	0.6977	0.2312	0.055*	
C20	−0.3444 (5)	0.6927 (4)	0.1289 (4)	0.0451 (13)	
H20	−0.352	0.7756	0.1116	0.054*	
C21	−0.2375 (5)	0.6174 (4)	0.0834 (4)	0.0447 (13)	
H21	−0.1705	0.6484	0.033	0.054*	
C22	−0.2254 (4)	0.4967 (4)	0.1096 (3)	0.0359 (11)	
H22	−0.1502	0.4469	0.0753	0.043*	
C23	−0.1523 (4)	0.2334 (3)	0.1934 (3)	0.0264 (10)	
C24	−0.0855 (4)	0.2006 (3)	0.2661 (3)	0.0302 (10)	
H24	−0.1288	0.2086	0.3379	0.036*	
C25	0.0425 (4)	0.1567 (4)	0.2370 (3)	0.0336 (11)	
H25	0.0847	0.1352	0.2888	0.04*	
C26	0.1079 (4)	0.1444 (4)	0.1341 (3)	0.0349 (11)	
H26	0.1954	0.1161	0.1139	0.042*	
C27	0.0451 (4)	0.1735 (4)	0.0602 (4)	0.0364 (11)	
H27	0.0893	0.1643	−0.0112	0.044*	
C28	−0.0825 (4)	0.2163 (3)	0.0902 (3)	0.0322 (11)	
H28	−0.1241	0.2346	0.0385	0.039*	
C29	−0.3729 (3)	0.2624 (3)	0.3513 (3)	0.0270 (10)	
C30	−0.4455 (4)	0.1817 (4)	0.4011 (3)	0.0315 (11)	
H30	−0.4562	0.1443	0.3571	0.038*	
C31	−0.5026 (3)	0.1534 (4)	0.5111 (3)	0.0332 (11)	
H31	−0.5502	0.0972	0.5407	0.04*	
C32	−0.4909 (4)	0.2062 (4)	0.5778 (4)	0.0346 (11)	
H32	−0.5306	0.1877	0.6532	0.042*	
C33	−0.4207 (4)	0.2862 (4)	0.5334 (3)	0.0353 (11)	
H33	−0.4107	0.3229	0.5783	0.042*	
C34	−0.3642 (4)	0.3138 (4)	0.4228 (3)	0.0321 (11)	
H34	−0.3172	0.3705	0.3942	0.038*	
C35	−0.3633 (3)	0.2597 (4)	0.1567 (3)	0.0283 (10)	
C36	−0.3433 (4)	0.1403 (4)	0.1619 (3)	0.0354 (11)	
H36	−0.2868	0.0826	0.1985	0.042*	
C37	−0.4004 (4)	0.1000 (4)	0.1170 (3)	0.0379 (12)	
H37	−0.384	0.0171	0.1242	0.046*	
C38	−0.4817 (4)	0.1818 (4)	0.0615 (3)	0.0385 (12)	
H38	−0.5229	0.1559	0.0312	0.046*	
C39	−0.5019 (4)	0.3019 (4)	0.0509 (3)	0.0349 (11)	
H39	−0.556	0.3592	0.0116	0.042*	
C40	−0.4439 (3)	0.3389 (4)	0.0972 (3)	0.0289 (10)	
H40	−0.4593	0.422	0.0883	0.035*	
N1	0.0866 (3)	0.2692 (3)	0.4410 (3)	0.0294 (8)	
N2	0.1900 (3)	0.4352 (3)	0.2776 (3)	0.0259 (8)	
N3	−0.0579 (3)	0.4664 (3)	0.3011 (3)	0.0391 (10)	
H3A	−0.0747	0.3944	0.3236	0.047*	
H3B	−0.1303	0.5243	0.3031	0.047*	
N4	0.0413 (3)	0.6636 (3)	0.2796 (2)	0.0280 (8)	
O1	0.1119 (2)	0.4845 (2)	0.4840 (2)	0.0298 (7)	
B1	−0.3033 (4)	0.2995 (4)	0.2217 (4)	0.0271 (12)	
Mn1	0.02600 (6)	0.47824 (6)	0.40381 (5)	0.0301 (2)	
O2	0.3085 (5)	0.4792 (4)	0.5293 (7)	0.095 (3)	0.870 (12)
H2O	0.2516	0.4701	0.5198	0.143*	0.870 (12)
H2P	0.362	0.4187	0.5228	0.143*	0.870 (12)
O2B	0.347 (4)	0.485 (3)	0.442 (5)	0.095 (3)	0.130 (12)
H2Q	0.2976	0.4569	0.4412	0.143*	0.130 (12)
H2R	0.4128	0.4381	0.4254	0.143*	0.130 (12)

### Di-µ-oxido-bis{[N,N-bis(6-methyl-2-pyridilmethyl)ethane-1,2-diamine]manganese(II)}(Mn—Mn) bis(tetraphenylborate) dihydrate (Complex1) Atomic displacement parameters (Å^2^)

**Table:**

	*U*^11^	*U*^22^	*U*^33^	*U*^12^	*U*^13^	*U*^23^
C1	0.031 (2)	0.033 (3)	0.022 (2)	−0.010 (2)	−0.008 (2)	−0.007 (2)
C2	0.026 (2)	0.036 (3)	0.030 (3)	−0.011 (2)	0.002 (2)	−0.009 (2)
C3	0.032 (2)	0.027 (2)	0.035 (3)	−0.012 (2)	−0.007 (2)	−0.001 (2)
C4	0.027 (2)	0.030 (2)	0.041 (3)	−0.005 (2)	−0.010 (2)	−0.005 (2)
C5	0.025 (2)	0.028 (3)	0.040 (3)	−0.001 (2)	−0.009 (2)	−0.010 (2)
C6	0.021 (2)	0.031 (2)	0.028 (2)	−0.0078 (19)	−0.0009 (19)	−0.0061 (19)
C7	0.023 (2)	0.034 (3)	0.024 (2)	−0.003 (2)	0.001 (2)	−0.0071 (19)
C8	0.030 (2)	0.036 (3)	0.030 (3)	−0.006 (2)	−0.003 (2)	−0.008 (2)
C9	0.033 (3)	0.038 (3)	0.033 (3)	−0.009 (2)	−0.008 (2)	−0.006 (2)
C10	0.021 (2)	0.032 (3)	0.032 (2)	−0.0058 (19)	0.002 (2)	−0.006 (2)
C11	0.028 (2)	0.026 (2)	0.023 (2)	−0.005 (2)	0.001 (2)	−0.0075 (19)
C12	0.028 (2)	0.032 (3)	0.028 (2)	−0.008 (2)	0.003 (2)	−0.008 (2)
C13	0.043 (3)	0.030 (3)	0.027 (3)	−0.012 (2)	0.004 (2)	−0.007 (2)
C14	0.040 (3)	0.031 (3)	0.022 (2)	−0.003 (2)	0.001 (2)	−0.006 (2)
C15	0.027 (2)	0.037 (3)	0.025 (2)	−0.004 (2)	−0.002 (2)	−0.008 (2)
C16	0.025 (2)	0.036 (3)	0.040 (3)	0.001 (2)	−0.004 (2)	−0.008 (2)
C17	0.032 (2)	0.031 (2)	0.025 (2)	−0.006 (2)	−0.013 (2)	−0.004 (2)
C18	0.038 (3)	0.034 (3)	0.027 (2)	−0.009 (2)	−0.009 (2)	−0.005 (2)
C19	0.062 (3)	0.039 (3)	0.033 (3)	0.001 (3)	−0.019 (3)	−0.014 (2)
C20	0.073 (4)	0.030 (3)	0.042 (3)	−0.017 (3)	−0.027 (3)	−0.005 (2)
C21	0.051 (3)	0.039 (3)	0.047 (3)	−0.018 (3)	−0.019 (3)	−0.001 (2)
C22	0.036 (3)	0.032 (3)	0.036 (3)	−0.013 (2)	−0.011 (2)	−0.001 (2)
C23	0.028 (2)	0.023 (2)	0.027 (2)	−0.0116 (19)	−0.006 (2)	−0.0006 (19)
C24	0.026 (2)	0.028 (2)	0.028 (2)	−0.008 (2)	−0.003 (2)	−0.002 (2)
C25	0.034 (3)	0.030 (2)	0.031 (3)	−0.011 (2)	−0.009 (2)	0.001 (2)
C26	0.025 (2)	0.035 (3)	0.037 (3)	−0.012 (2)	−0.001 (2)	−0.005 (2)
C27	0.032 (3)	0.035 (3)	0.033 (3)	−0.013 (2)	0.002 (2)	−0.008 (2)
C28	0.032 (2)	0.032 (3)	0.028 (3)	−0.008 (2)	−0.008 (2)	−0.004 (2)
C29	0.020 (2)	0.027 (2)	0.028 (2)	0.0006 (18)	−0.007 (2)	−0.006 (2)
C30	0.024 (2)	0.031 (2)	0.032 (3)	−0.005 (2)	−0.008 (2)	−0.002 (2)
C31	0.020 (2)	0.033 (3)	0.034 (3)	−0.0029 (19)	−0.003 (2)	−0.004 (2)
C32	0.021 (2)	0.040 (3)	0.029 (3)	−0.003 (2)	0.002 (2)	−0.009 (2)
C33	0.029 (2)	0.042 (3)	0.027 (3)	−0.003 (2)	−0.001 (2)	−0.012 (2)
C34	0.024 (2)	0.035 (3)	0.031 (3)	−0.007 (2)	−0.004 (2)	−0.006 (2)
C35	0.018 (2)	0.033 (3)	0.022 (2)	−0.0097 (19)	0.0077 (19)	−0.0051 (19)
C36	0.031 (2)	0.035 (3)	0.037 (3)	−0.011 (2)	−0.010 (2)	−0.003 (2)
C37	0.039 (3)	0.032 (3)	0.039 (3)	−0.010 (2)	−0.010 (2)	−0.006 (2)
C38	0.036 (3)	0.052 (3)	0.033 (3)	−0.022 (2)	0.000 (2)	−0.017 (2)
C39	0.025 (2)	0.046 (3)	0.025 (2)	−0.005 (2)	−0.001 (2)	−0.010 (2)
C40	0.024 (2)	0.030 (2)	0.023 (2)	−0.003 (2)	0.002 (2)	−0.009 (2)
N1	0.0274 (19)	0.029 (2)	0.0246 (19)	−0.0081 (16)	−0.0014 (17)	−0.0049 (16)
N2	0.0218 (18)	0.0237 (19)	0.0247 (19)	−0.0028 (15)	−0.0039 (16)	−0.0040 (16)
N3	0.027 (2)	0.028 (2)	0.044 (2)	−0.0044 (16)	0.0006 (19)	−0.0038 (17)
N4	0.0206 (18)	0.031 (2)	0.0209 (19)	−0.0032 (16)	0.0021 (16)	−0.0062 (16)
O1	0.0189 (14)	0.0322 (17)	0.0293 (16)	−0.0051 (13)	0.0014 (13)	−0.0084 (13)
B1	0.024 (3)	0.029 (3)	0.025 (3)	−0.007 (2)	−0.005 (2)	−0.005 (2)
Mn1	0.0197 (3)	0.0288 (4)	0.0290 (4)	−0.0030 (3)	0.0022 (3)	−0.0064 (3)
O2	0.063 (3)	0.082 (3)	0.163 (8)	0.007 (3)	−0.046 (4)	−0.068 (4)
O2B	0.063 (3)	0.082 (3)	0.163 (8)	0.007 (3)	−0.046 (4)	−0.068 (4)

### Di-µ-oxido-bis{[N,N-bis(6-methyl-2-pyridilmethyl)ethane-1,2-diamine]manganese(II)}(Mn—Mn) bis(tetraphenylborate) dihydrate (Complex1) Geometric parameters (Å, º)

**Table:**

C1—N1	1.353 (5)	C22—H22	0.95
C1—C3	1.389 (5)	C23—C24	1.396 (5)
C1—C2	1.496 (5)	C23—C28	1.397 (5)
C2—H2A	0.98	C23—B1	1.666 (6)
C2—H2D	0.98	C24—C25	1.392 (5)
C2—H2C	0.98	C24—H24	0.95
C3—C4	1.371 (5)	C25—C26	1.369 (6)
C3—H3	0.95	C25—H25	0.95
C4—C5	1.379 (5)	C26—C27	1.381 (6)
C4—H4	0.95	C26—H26	0.95
C5—C6	1.361 (5)	C27—C28	1.387 (5)
C5—H5	0.95	C27—H27	0.95
C6—N1	1.370 (5)	C28—H28	0.95
C6—C7	1.499 (5)	C29—C30	1.398 (5)
C7—N2	1.487 (5)	C29—C34	1.401 (6)
C7—H7A	0.99	C29—B1	1.640 (6)
C7—H7B	0.99	C30—C31	1.386 (5)
C8—N2	1.493 (5)	C30—H30	0.95
C8—C9	1.525 (5)	C31—C32	1.374 (6)
C8—H8A	0.99	C31—H31	0.95
C8—H8B	0.99	C32—C33	1.372 (5)
C9—N3	1.492 (5)	C32—H32	0.95
C9—H9A	0.99	C33—C34	1.389 (5)
C9—H9B	0.99	C33—H33	0.95
C10—N2	1.479 (5)	C34—H34	0.95
C10—C11	1.504 (5)	C35—C36	1.392 (5)
C10—H10A	0.99	C35—C40	1.403 (5)
C10—H10B	0.99	C35—B1	1.631 (6)
C11—N4	1.356 (4)	C36—C37	1.385 (6)
C11—C12	1.368 (5)	C36—H36	0.95
C12—C13	1.391 (5)	C37—C38	1.384 (6)
C12—H12	0.95	C37—H37	0.95
C13—C14	1.374 (5)	C38—C39	1.382 (6)
C13—H13	0.95	C38—H38	0.95
C14—C15	1.376 (5)	C39—C40	1.382 (6)
C14—H14	0.95	C39—H39	0.95
C15—N4	1.354 (5)	C40—H40	0.95
C15—C16	1.501 (5)	N1—Mn1	2.348 (3)
C16—H16A	0.98	N2—Mn1	2.123 (3)
C16—H16B	0.98	N3—Mn1	2.111 (4)
C16—H16C	0.98	N3—H3A	0.91
C17—C18	1.399 (5)	N3—H3B	0.91
C17—C22	1.405 (5)	N4—Mn1	2.368 (3)
C17—B1	1.642 (6)	O1—Mn1	1.829 (3)
C18—C19	1.390 (6)	O1—Mn1^i^	1.835 (2)
C18—H18	0.95	Mn1—O1^i^	1.835 (2)
C19—C20	1.385 (6)	Mn1—Mn1^i^	2.6899 (15)
C19—H19	0.95	O2—H2O	0.8037
C20—C21	1.362 (6)	O2—H2P	0.8012
C20—H20	0.95	O2B—H2Q	0.8011
C21—C22	1.378 (6)	O2B—H2R	0.8066
C21—H21	0.95		
			
N1—C1—C3	120.3 (4)	C25—C26—C27	119.2 (4)
N1—C1—C2	118.0 (4)	C25—C26—H26	120.4
C3—C1—C2	121.7 (4)	C27—C26—H26	120.4
C1—C2—H2A	109.5	C26—C27—C28	119.9 (4)
C1—C2—H2D	109.5	C26—C27—H27	120
H2A—C2—H2D	109.5	C28—C27—H27	120
C1—C2—H2C	109.5	C27—C28—C23	122.7 (4)
H2A—C2—H2C	109.5	C27—C28—H28	118.7
H2D—C2—H2C	109.5	C23—C28—H28	118.7
C4—C3—C1	120.8 (4)	C30—C29—C34	113.9 (4)
C4—C3—H3	119.6	C30—C29—B1	125.5 (4)
C1—C3—H3	119.6	C34—C29—B1	120.6 (4)
C3—C4—C5	118.4 (4)	C31—C30—C29	123.4 (4)
C3—C4—H4	120.8	C31—C30—H30	118.3
C5—C4—H4	120.8	C29—C30—H30	118.3
C6—C5—C4	119.7 (4)	C32—C31—C30	120.4 (4)
C6—C5—H5	120.2	C32—C31—H31	119.8
C4—C5—H5	120.2	C30—C31—H31	119.8
C5—C6—N1	122.2 (4)	C33—C32—C31	118.8 (4)
C5—C6—C7	122.7 (4)	C33—C32—H32	120.6
N1—C6—C7	115.0 (3)	C31—C32—H32	120.6
N2—C7—C6	112.1 (3)	C32—C33—C34	120.1 (4)
N2—C7—H7A	109.2	C32—C33—H33	119.9
C6—C7—H7A	109.2	C34—C33—H33	119.9
N2—C7—H7B	109.2	C33—C34—C29	123.5 (4)
C6—C7—H7B	109.2	C33—C34—H34	118.3
H7A—C7—H7B	107.9	C29—C34—H34	118.3
N2—C8—C9	113.1 (3)	C36—C35—C40	114.1 (4)
N2—C8—H8A	108.9	C36—C35—B1	121.4 (4)
C9—C8—H8A	108.9	C40—C35—B1	124.4 (4)
N2—C8—H8B	108.9	C37—C36—C35	124.2 (4)
C9—C8—H8B	108.9	C37—C36—H36	117.9
H8A—C8—H8B	107.8	C35—C36—H36	117.9
N3—C9—C8	108.6 (3)	C38—C37—C36	119.3 (4)
N3—C9—H9A	110	C38—C37—H37	120.4
C8—C9—H9A	110	C36—C37—H37	120.4
N3—C9—H9B	110	C39—C38—C37	118.9 (4)
C8—C9—H9B	110	C39—C38—H38	120.6
H9A—C9—H9B	108.3	C37—C38—H38	120.6
N2—C10—C11	112.7 (3)	C40—C39—C38	120.3 (4)
N2—C10—H10A	109	C40—C39—H39	119.9
C11—C10—H10A	109	C38—C39—H39	119.9
N2—C10—H10B	109	C39—C40—C35	123.2 (4)
C11—C10—H10B	109	C39—C40—H40	118.4
H10A—C10—H10B	107.8	C35—C40—H40	118.4
N4—C11—C12	123.2 (4)	C1—N1—C6	118.4 (3)
N4—C11—C10	116.1 (3)	C1—N1—Mn1	130.7 (3)
C12—C11—C10	120.6 (4)	C6—N1—Mn1	110.5 (2)
C11—C12—C13	118.5 (4)	C10—N2—C7	108.6 (3)
C11—C12—H12	120.8	C10—N2—C8	113.0 (3)
C13—C12—H12	120.8	C7—N2—C8	109.7 (3)
C14—C13—C12	118.6 (4)	C10—N2—Mn1	107.9 (2)
C14—C13—H13	120.7	C7—N2—Mn1	108.2 (2)
C12—C13—H13	120.7	C8—N2—Mn1	109.3 (2)
C13—C14—C15	120.6 (4)	C9—N3—Mn1	109.4 (3)
C13—C14—H14	119.7	C9—N3—H3A	109.8
C15—C14—H14	119.7	Mn1—N3—H3A	109.8
N4—C15—C14	121.0 (4)	C9—N3—H3B	109.8
N4—C15—C16	116.9 (4)	Mn1—N3—H3B	109.8
C14—C15—C16	122.1 (4)	H3A—N3—H3B	108.2
C15—C16—H16A	109.5	C15—N4—C11	118.0 (3)
C15—C16—H16B	109.5	C15—N4—Mn1	132.6 (3)
H16A—C16—H16B	109.5	C11—N4—Mn1	109.3 (2)
C15—C16—H16C	109.5	Mn1—O1—Mn1^i^	94.47 (12)
H16A—C16—H16C	109.5	C35—B1—C29	109.0 (3)
H16B—C16—H16C	109.5	C35—B1—C17	111.5 (4)
C18—C17—C22	114.7 (4)	C29—B1—C17	108.8 (3)
C18—C17—B1	121.9 (4)	C35—B1—C23	109.3 (3)
C22—C17—B1	123.4 (4)	C29—B1—C23	111.2 (3)
C19—C18—C17	122.5 (4)	C17—B1—C23	107.1 (3)
C19—C18—H18	118.7	O1—Mn1—O1^i^	85.53 (12)
C17—C18—H18	118.7	O1—Mn1—N3	174.90 (12)
C20—C19—C18	120.2 (4)	O1^i^—Mn1—N3	99.56 (13)
C20—C19—H19	119.9	O1—Mn1—N2	92.13 (12)
C18—C19—H19	119.9	O1^i^—Mn1—N2	177.66 (13)
C21—C20—C19	118.8 (4)	N3—Mn1—N2	82.78 (13)
C21—C20—H20	120.6	O1—Mn1—N1	93.76 (12)
C19—C20—H20	120.6	O1^i^—Mn1—N1	105.29 (11)
C20—C21—C22	120.7 (4)	N3—Mn1—N1	84.68 (12)
C20—C21—H21	119.6	N2—Mn1—N1	74.79 (11)
C22—C21—H21	119.6	O1—Mn1—N4	95.77 (12)
C21—C22—C17	123.0 (4)	O1^i^—Mn1—N4	104.76 (11)
C21—C22—H22	118.5	N3—Mn1—N4	83.23 (12)
C17—C22—H22	118.5	N2—Mn1—N4	75.49 (11)
C24—C23—C28	115.5 (4)	N1—Mn1—N4	149.05 (11)
C24—C23—B1	123.3 (4)	O1—Mn1—Mn1^i^	42.86 (8)
C28—C23—B1	121.0 (4)	O1^i^—Mn1—Mn1^i^	42.67 (9)
C25—C24—C23	122.3 (4)	N3—Mn1—Mn1^i^	142.23 (10)
C25—C24—H24	118.9	N2—Mn1—Mn1^i^	134.99 (10)
C23—C24—H24	118.9	N1—Mn1—Mn1^i^	102.97 (9)
C26—C25—C24	120.4 (4)	N4—Mn1—Mn1^i^	104.02 (9)
C26—C25—H25	119.8	H2O—O2—H2P	104.8
C24—C25—H25	119.8	H2Q—O2B—H2R	105

Symmetry code: (i) −*x*, −*y*+1, −*z*+1.

### Di-µ-oxido-bis{[N,N-bis(6-methyl-2-pyridilmethyl)propane-1,3-diamine]manganese(II)}(Mn—Mn) bis(tetraphenylborate) diethyl ether disolvate (Complex2) Crystal data

**Table:**

[Mn(C_17_H_24_N_4_)_2_O_2_](C_24_H_20_B)_2_·2C_4_H_10_O	*F*(000) = 1592
*M**_r_* = 1497.34	*D*_x_ = 1.286 Mg m^−^^3^
Monoclinic, *P*2_1_/*n*	Mo *K*α radiation, λ = 0.71073 Å
Hall symbol: -P 2yn	Cell parameters from 110 reflections
*a* = 15.9472 (16) Å	θ = 3–20°
*b* = 13.8380 (14) Å	µ = 0.39 mm^−^^1^
*c* = 17.5219 (17) Å	*T* = 100 K
β = 91.123 (5)°	Plate, purple
*V* = 3865.9 (7) Å^3^	0.1 × 0.05 × 0.05 mm
*Z* = 2	

### Di-µ-oxido-bis{[N,N-bis(6-methyl-2-pyridilmethyl)propane-1,3-diamine]manganese(II)}(Mn—Mn) bis(tetraphenylborate) diethyl ether disolvate (Complex2) Data collection

**Table:**

Bruker APEXII CCD area-detector diffractometer	9679 independent reflections
Radiation source: fine-focus sealed tube	7420 reflections with *I* > 2σ(*I*)
Graphite monochromator	*R*_int_ = 0.068
φ and ω scans	θ_max_ = 28.5°, θ_min_ = 1.7°
Absorption correction: multi-scan (SADABS; Bruker, 2007)	*h* = −21→21
*T*_min_ = 0.915, *T*_max_ = 0.947	*k* = −18→18
138191 measured reflections	*l* = −23→23

### Di-µ-oxido-bis{[N,N-bis(6-methyl-2-pyridilmethyl)propane-1,3-diamine]manganese(II)}(Mn—Mn) bis(tetraphenylborate) diethyl ether disolvate (Complex2) Refinement

**Table:**

Refinement on *F*^2^	Primary atom site location: structure-invariant direct methods
Least-squares matrix: full	Secondary atom site location: difference Fourier map
*R*[*F*^2^ > 2σ(*F*^2^)] = 0.049	Hydrogen site location: inferred from neighbouring sites
*wR*(*F*^2^) = 0.134	H-atom parameters constrained
*S* = 1.05	*w* = 1/[σ^2^(*F*_o_^2^) + (0.0542*P*)^2^ + 5.0764*P*] where *P* = (*F*_o_^2^ + 2*F*_c_^2^)/3
9679 reflections	(Δ/σ)_max_ = 0.004
517 parameters	Δρ_max_ = 0.66 e Å^−^^3^
29 restraints	Δρ_min_ = −1.01 e Å^−^^3^

### Di-µ-oxido-bis{[N,N-bis(6-methyl-2-pyridilmethyl)propane-1,3-diamine]manganese(II)}(Mn—Mn) bis(tetraphenylborate) diethyl ether disolvate (Complex2) Special details

**Table:**

Experimental. 20 seconds exposure, 0.5 degree steps, 40mm distance
Geometry. All esds (except the esd in the dihedral angle between two l.s. planes) are estimated using the full covariance matrix. The cell esds are taken into account individually in the estimation of esds in distances, angles and torsion angles; correlations between esds in cell parameters are only used when they are defined by crystal symmetry. An approximate (isotropic) treatment of cell esds is used for estimating esds involving l.s. planes.

### Di-µ-oxido-bis{[N,N-bis(6-methyl-2-pyridilmethyl)propane-1,3-diamine]manganese(II)}(Mn—Mn) bis(tetraphenylborate) diethyl ether disolvate (Complex2) Fractional atomic coordinates and isotropic or equivalent isotropic displacement parameters (Å^2^)

**Table:**

	*x*	*y*	*z*	*U*_iso_*/*U*_eq_	Occ. (<1)
N3	0.5515 (5)	0.7880 (5)	0.4465 (3)	0.0164 (8)	0.804 (5)
H1A	0.498717	0.771071	0.431004	0.02*	0.804 (5)
H1B	0.580243	0.803082	0.403801	0.02*	0.804 (5)
C1	0.5915 (2)	0.7006 (3)	0.4806 (2)	0.0218 (8)	0.804 (5)
H1C	0.603548	0.65384	0.439551	0.026*	0.804 (5)
H1D	0.55183	0.669739	0.515896	0.026*	0.804 (5)
C2	0.67233 (16)	0.72410 (19)	0.52387 (16)	0.0205 (6)	0.804 (5)
H2A	0.705893	0.664216	0.529238	0.025*	0.804 (5)
H2B	0.705093	0.770383	0.493344	0.025*	0.804 (5)
C3	0.6596 (7)	0.7667 (5)	0.6026 (4)	0.0194 (10)	0.804 (5)
H3A	0.714296	0.766217	0.630293	0.023*	0.804 (5)
H3B	0.621377	0.723634	0.630579	0.023*	0.804 (5)
N3B	0.552 (2)	0.791 (2)	0.4623 (14)	0.020 (3)	0.196 (5)
H1B1	0.501206	0.761127	0.468206	0.024*	0.196 (5)
H1B2	0.555456	0.803809	0.411561	0.024*	0.196 (5)
C1B	0.6166 (11)	0.7148 (15)	0.4790 (9)	0.023 (3)	0.196 (5)
H1B3	0.600277	0.653562	0.453674	0.028*	0.196 (5)
H1B4	0.671279	0.735517	0.458779	0.028*	0.196 (5)
C2B	0.6246 (7)	0.6993 (8)	0.5636 (6)	0.023 (2)	0.196 (5)
H2B1	0.656439	0.638984	0.573199	0.028*	0.196 (5)
H2B2	0.567863	0.690589	0.584478	0.028*	0.196 (5)
C3B	0.668 (3)	0.781 (3)	0.6058 (19)	0.018 (3)	0.196 (5)
H3B1	0.677929	0.761392	0.659468	0.021*	0.196 (5)
H3B2	0.723357	0.792265	0.582827	0.021*	0.196 (5)
C4	0.69163 (14)	0.94311 (17)	0.60980 (12)	0.0214 (4)	
H4A	0.736762	0.920165	0.644915	0.026*	
H4B	0.668641	1.003576	0.631168	0.026*	
C5	0.72837 (13)	0.96430 (15)	0.53303 (12)	0.0184 (4)	
C6	0.80978 (13)	0.99839 (16)	0.52841 (13)	0.0214 (4)	
H6	0.844797	1.003335	0.57273	0.026*	
C7	0.83877 (14)	1.02508 (18)	0.45742 (13)	0.0256 (5)	
H7	0.894135	1.049312	0.452456	0.031*	
C8	0.78671 (14)	1.01622 (18)	0.39401 (13)	0.0251 (5)	
H8	0.805983	1.033676	0.344935	0.03*	
C9	0.70530 (14)	0.98125 (16)	0.40284 (12)	0.0199 (4)	
C10	0.64684 (14)	0.97137 (18)	0.33522 (12)	0.0239 (5)	
H10A	0.657755	0.910092	0.309193	0.036*	
H10B	0.655828	1.025123	0.299904	0.036*	
H10C	0.588727	0.972421	0.352384	0.036*	
C11	0.57251 (13)	0.87692 (16)	0.67526 (12)	0.0198 (4)	
H11A	0.561089	0.945784	0.686312	0.024*	
H11B	0.603786	0.849279	0.719476	0.024*	
C12	0.49079 (13)	0.82365 (16)	0.66401 (12)	0.0199 (4)	
C13	0.45371 (14)	0.77419 (17)	0.72304 (13)	0.0248 (5)	
H13	0.481183	0.768084	0.7714	0.03*	
C14	0.37523 (15)	0.73375 (18)	0.70960 (14)	0.0280 (5)	
H14	0.347712	0.699918	0.749089	0.034*	
C15	0.33745 (14)	0.74296 (17)	0.63861 (14)	0.0254 (5)	
H15	0.283211	0.716622	0.62923	0.03*	
C16	0.37914 (13)	0.79116 (15)	0.58043 (13)	0.0206 (4)	
C17	0.34223 (14)	0.79910 (17)	0.50155 (13)	0.0237 (5)	
H17A	0.368006	0.853577	0.475041	0.036*	
H17B	0.281614	0.809664	0.50447	0.036*	
H17C	0.352892	0.73926	0.473439	0.036*	
C18	0.4398 (3)	0.8992 (4)	0.9245 (2)	0.0780 (13)	
H18A	0.460819	0.84242	0.897422	0.117*	
H18B	0.473517	0.909603	0.971087	0.117*	
H18C	0.381116	0.888576	0.93787	0.117*	
C19	0.4452 (3)	0.9794 (3)	0.8780 (2)	0.0687 (11)	
H19A	0.411612	0.969258	0.830579	0.082*	
H19B	0.504297	0.99035	0.863897	0.082*	
O2	0.4151 (2)	1.0594 (3)	0.9173 (2)	0.0911 (10)	
C20	0.4051 (2)	1.1363 (3)	0.87248 (19)	0.0541 (8)	
H20A	0.356626	1.127091	0.837118	0.065*	
H20B	0.455934	1.147018	0.841994	0.065*	
C21	0.3904 (3)	1.2211 (5)	0.9244 (3)	0.0941 (18)	
H21A	0.382645	1.279793	0.89372	0.141*	
H21B	0.339989	1.209485	0.954214	0.141*	
H21C	0.438815	1.229316	0.959066	0.141*	
C22	0.09092 (13)	1.17753 (16)	0.28704 (13)	0.0211 (4)	
C23	0.11769 (14)	1.26414 (17)	0.25359 (14)	0.0260 (5)	
H23	0.120968	1.267259	0.199567	0.031*	
C24	0.13964 (14)	1.34558 (18)	0.29605 (16)	0.0299 (5)	
H24	0.157021	1.402795	0.270913	0.036*	
C25	0.13615 (15)	1.34325 (18)	0.37494 (16)	0.0312 (6)	
H25	0.15186	1.39828	0.404316	0.037*	
C26	0.10935 (15)	1.25932 (18)	0.41053 (15)	0.0287 (5)	
H26	0.106439	1.256713	0.464594	0.034*	
C27	0.08681 (14)	1.17918 (17)	0.36692 (14)	0.0243 (5)	
H27	0.067748	1.122939	0.392392	0.029*	
C28	0.11586 (13)	1.07869 (15)	0.15746 (12)	0.0193 (4)	
C29	0.08354 (14)	1.05345 (16)	0.08536 (13)	0.0214 (4)	
H29	0.024784	1.043898	0.079414	0.026*	
C30	0.13412 (15)	1.04168 (17)	0.02163 (13)	0.0243 (5)	
H30	0.109362	1.025021	−0.026343	0.029*	
C31	0.21998 (15)	1.05422 (16)	0.02824 (14)	0.0254 (5)	
H31	0.25469	1.045107	−0.014614	0.03*	
C32	0.25456 (14)	1.08035 (16)	0.09852 (14)	0.0240 (5)	
H32	0.313397	1.089679	0.103891	0.029*	
C33	0.20347 (14)	1.09291 (17)	0.16106 (13)	0.0231 (4)	
H33	0.228616	1.111901	0.208321	0.028*	
C34	−0.04087 (13)	1.09627 (15)	0.21570 (12)	0.0187 (4)	
C35	−0.08489 (13)	1.18282 (17)	0.22570 (13)	0.0220 (4)	
H35	−0.054865	1.23814	0.243101	0.026*	
C36	−0.17108 (14)	1.19132 (18)	0.21119 (14)	0.0260 (5)	
H36	−0.198487	1.251277	0.219402	0.031*	
C37	−0.21652 (14)	1.11247 (18)	0.18487 (13)	0.0252 (5)	
H37	−0.275004	1.117859	0.174366	0.03*	
C38	−0.17535 (14)	1.02566 (17)	0.17411 (12)	0.0227 (5)	
H38	−0.205828	0.97085	0.156357	0.027*	
C39	−0.08937 (14)	1.01809 (16)	0.18916 (12)	0.0203 (4)	
H39	−0.062612	0.957703	0.181142	0.024*	
C40	0.07372 (13)	0.98213 (15)	0.28312 (12)	0.0178 (4)	
C41	0.01626 (13)	0.95352 (16)	0.33934 (12)	0.0202 (4)	
H41	−0.032073	0.992264	0.346761	0.024*	
C42	0.02706 (14)	0.87168 (17)	0.38412 (12)	0.0231 (5)	
H42	−0.013144	0.855906	0.42143	0.028*	
C43	0.09664 (15)	0.81266 (17)	0.37452 (13)	0.0243 (5)	
H43	0.105179	0.757155	0.405609	0.029*	
C44	0.15325 (14)	0.83664 (17)	0.31857 (13)	0.0241 (5)	
H44	0.200508	0.796391	0.310476	0.029*	
C45	0.14154 (13)	0.91924 (16)	0.27402 (12)	0.0209 (4)	
H45	0.181262	0.933416	0.235902	0.025*	
N2	0.62427 (11)	0.86938 (13)	0.60570 (10)	0.0177 (3)	
N4	0.67697 (11)	0.95591 (13)	0.47178 (10)	0.0174 (3)	
N1	0.45540 (11)	0.83090 (13)	0.59378 (10)	0.0186 (4)	
O1	0.47256 (9)	0.96773 (10)	0.43771 (8)	0.0173 (3)	
B1	0.06033 (14)	1.08398 (18)	0.23509 (14)	0.0183 (4)	
Mn1	0.54141 (2)	0.91694 (2)	0.51245 (2)	0.01497 (9)	

### Di-µ-oxido-bis{[N,N-bis(6-methyl-2-pyridilmethyl)propane-1,3-diamine]manganese(II)}(Mn—Mn) bis(tetraphenylborate) diethyl ether disolvate (Complex2) Atomic displacement parameters (Å^2^)

**Table:**

	*U*^11^	*U*^22^	*U*^33^	*U*^12^	*U*^13^	*U*^23^
N3	0.0174 (11)	0.0186 (12)	0.013 (2)	0.0024 (10)	−0.0015 (15)	−0.0061 (15)
C1	0.022 (2)	0.0159 (18)	0.0272 (14)	−0.0004 (15)	−0.0027 (14)	−0.0024 (11)
C2	0.0167 (13)	0.0180 (13)	0.0269 (14)	0.0031 (10)	0.0009 (10)	−0.0024 (10)
C3	0.018 (3)	0.016 (3)	0.0236 (15)	0.0048 (18)	−0.0020 (14)	0.0014 (15)
N3B	0.022 (4)	0.026 (4)	0.012 (6)	−0.002 (4)	0.000 (5)	0.001 (5)
C1B	0.024 (6)	0.015 (5)	0.031 (4)	0.000 (5)	0.003 (5)	−0.002 (4)
C2B	0.018 (4)	0.020 (4)	0.032 (4)	0.006 (3)	0.002 (3)	0.001 (3)
C3B	0.016 (5)	0.017 (6)	0.020 (4)	−0.001 (5)	−0.001 (4)	−0.002 (4)
C4	0.0188 (10)	0.0266 (11)	0.0187 (10)	−0.0047 (9)	−0.0032 (8)	0.0012 (8)
C5	0.0163 (10)	0.0179 (10)	0.0209 (10)	0.0015 (8)	−0.0004 (8)	−0.0001 (8)
C6	0.0157 (10)	0.0251 (11)	0.0233 (10)	0.0007 (8)	−0.0025 (8)	−0.0013 (9)
C7	0.0167 (10)	0.0322 (13)	0.0281 (12)	−0.0029 (9)	0.0043 (9)	−0.0026 (10)
C8	0.0210 (11)	0.0335 (13)	0.0209 (11)	0.0003 (9)	0.0068 (8)	0.0001 (9)
C9	0.0201 (10)	0.0197 (10)	0.0200 (10)	0.0014 (8)	0.0028 (8)	−0.0020 (8)
C10	0.0237 (11)	0.0290 (12)	0.0190 (10)	−0.0003 (9)	0.0014 (8)	−0.0021 (9)
C11	0.0202 (10)	0.0232 (11)	0.0160 (9)	0.0008 (8)	0.0013 (8)	0.0016 (8)
C12	0.0187 (10)	0.0190 (10)	0.0220 (10)	0.0037 (8)	0.0029 (8)	0.0022 (8)
C13	0.0227 (11)	0.0281 (12)	0.0237 (11)	0.0043 (9)	0.0028 (9)	0.0069 (9)
C14	0.0237 (11)	0.0263 (12)	0.0344 (13)	0.0024 (9)	0.0105 (10)	0.0109 (10)
C15	0.0168 (10)	0.0230 (11)	0.0366 (13)	−0.0003 (9)	0.0034 (9)	0.0046 (10)
C16	0.0170 (10)	0.0147 (10)	0.0300 (11)	0.0027 (8)	0.0018 (8)	0.0000 (8)
C17	0.0180 (10)	0.0204 (11)	0.0325 (12)	−0.0022 (8)	−0.0019 (9)	−0.0003 (9)
C18	0.086 (3)	0.089 (3)	0.058 (2)	0.010 (3)	−0.017 (2)	−0.012 (2)
C19	0.071 (3)	0.068 (3)	0.066 (2)	−0.003 (2)	−0.014 (2)	−0.010 (2)
O2	0.077 (2)	0.082 (2)	0.115 (3)	−0.0006 (17)	0.0279 (19)	0.010 (2)
C20	0.056 (2)	0.067 (2)	0.0385 (17)	0.0003 (17)	−0.0055 (14)	0.0105 (16)
C21	0.058 (3)	0.161 (5)	0.064 (3)	−0.016 (3)	0.012 (2)	−0.032 (3)
C22	0.0125 (9)	0.0216 (11)	0.0293 (11)	0.0008 (8)	−0.0001 (8)	−0.0020 (9)
C23	0.0192 (11)	0.0269 (12)	0.0322 (12)	−0.0016 (9)	0.0045 (9)	−0.0019 (10)
C24	0.0192 (11)	0.0231 (12)	0.0475 (15)	−0.0020 (9)	0.0030 (10)	−0.0016 (10)
C25	0.0195 (11)	0.0234 (12)	0.0505 (16)	0.0003 (9)	−0.0063 (10)	−0.0114 (11)
C26	0.0237 (11)	0.0296 (13)	0.0325 (13)	0.0043 (10)	−0.0071 (9)	−0.0082 (10)
C27	0.0183 (10)	0.0218 (11)	0.0326 (12)	0.0015 (8)	−0.0032 (9)	−0.0024 (9)
C28	0.0157 (10)	0.0173 (10)	0.0250 (10)	0.0011 (8)	0.0024 (8)	0.0016 (8)
C29	0.0191 (10)	0.0194 (10)	0.0259 (11)	−0.0010 (8)	0.0015 (8)	0.0013 (8)
C30	0.0279 (12)	0.0211 (11)	0.0241 (11)	−0.0022 (9)	0.0032 (9)	−0.0019 (9)
C31	0.0281 (12)	0.0191 (11)	0.0293 (12)	0.0020 (9)	0.0100 (9)	0.0021 (9)
C32	0.0162 (10)	0.0225 (11)	0.0335 (12)	0.0018 (8)	0.0046 (9)	0.0038 (9)
C33	0.0179 (10)	0.0265 (12)	0.0250 (11)	−0.0005 (9)	−0.0002 (8)	0.0015 (9)
C34	0.0175 (10)	0.0208 (10)	0.0179 (10)	−0.0019 (8)	0.0019 (8)	0.0026 (8)
C35	0.0177 (10)	0.0225 (11)	0.0258 (11)	−0.0017 (8)	0.0021 (8)	0.0004 (9)
C36	0.0192 (11)	0.0266 (12)	0.0323 (12)	0.0042 (9)	0.0047 (9)	0.0039 (10)
C37	0.0144 (10)	0.0361 (13)	0.0252 (11)	−0.0003 (9)	0.0011 (8)	0.0059 (10)
C38	0.0181 (10)	0.0297 (12)	0.0202 (10)	−0.0063 (9)	0.0000 (8)	0.0039 (9)
C39	0.0191 (10)	0.0206 (10)	0.0214 (10)	−0.0008 (8)	0.0021 (8)	0.0021 (8)
C40	0.0153 (10)	0.0193 (10)	0.0187 (10)	−0.0017 (8)	−0.0018 (8)	−0.0026 (8)
C41	0.0171 (10)	0.0220 (10)	0.0216 (10)	−0.0004 (8)	0.0004 (8)	−0.0021 (8)
C42	0.0220 (11)	0.0275 (12)	0.0197 (10)	−0.0055 (9)	0.0006 (8)	−0.0010 (9)
C43	0.0291 (12)	0.0200 (11)	0.0235 (11)	−0.0005 (9)	−0.0057 (9)	0.0013 (9)
C44	0.0224 (11)	0.0220 (11)	0.0280 (11)	0.0043 (9)	−0.0008 (9)	−0.0034 (9)
C45	0.0179 (10)	0.0227 (11)	0.0220 (10)	0.0001 (8)	0.0005 (8)	−0.0032 (9)
N2	0.0160 (8)	0.0188 (9)	0.0182 (8)	−0.0001 (7)	0.0002 (7)	0.0013 (7)
N4	0.0157 (8)	0.0177 (8)	0.0187 (8)	0.0008 (7)	−0.0002 (6)	−0.0007 (7)
N1	0.0176 (8)	0.0166 (8)	0.0216 (9)	0.0009 (7)	0.0008 (7)	0.0013 (7)
O1	0.0174 (7)	0.0169 (7)	0.0173 (7)	0.0008 (6)	−0.0015 (5)	−0.0013 (6)
B1	0.0132 (10)	0.0192 (11)	0.0226 (11)	−0.0002 (9)	0.0013 (8)	−0.0014 (9)
Mn1	0.01475 (15)	0.01496 (15)	0.01515 (15)	0.00061 (12)	−0.00081 (11)	−0.00073 (11)

### Di-µ-oxido-bis{[N,N-bis(6-methyl-2-pyridilmethyl)propane-1,3-diamine]manganese(II)}(Mn—Mn) bis(tetraphenylborate) diethyl ether disolvate (Complex2) Geometric parameters (Å, º)

**Table:**

N3—C1	1.487 (5)	C19—O2	1.394 (5)
N3—Mn1	2.133 (6)	C19—H19A	0.99
N3—H1A	0.91	C19—H19B	0.99
N3—H1B	0.91	O2—C20	1.331 (5)
C1—C2	1.518 (4)	C20—C21	1.507 (6)
C1—H1C	0.99	C20—H20A	0.99
C1—H1D	0.99	C20—H20B	0.99
C2—C3	1.517 (8)	C21—H21A	0.98
C2—H2A	0.99	C21—H21B	0.98
C2—H2B	0.99	C21—H21C	0.98
C3—N2	1.529 (9)	C22—C27	1.403 (3)
C3—H3A	0.99	C22—C23	1.404 (3)
C3—H3B	0.99	C22—B1	1.651 (3)
N3B—C1B	1.502 (16)	C23—C24	1.391 (3)
N3B—Mn1	1.96 (3)	C23—H23	0.95
N3B—H1B1	0.91	C24—C25	1.385 (4)
N3B—H1B2	0.91	C24—H24	0.95
C1B—C2B	1.500 (15)	C25—C26	1.390 (4)
C1B—H1B3	0.99	C25—H25	0.95
C1B—H1B4	0.99	C26—C27	1.390 (3)
C2B—C3B	1.516 (18)	C26—H26	0.95
C2B—H2B1	0.99	C27—H27	0.95
C2B—H2B2	0.99	C28—C29	1.399 (3)
C3B—N2	1.40 (5)	C28—C33	1.411 (3)
C3B—H3B1	0.99	C28—B1	1.639 (3)
C3B—H3B2	0.99	C29—C30	1.400 (3)
C4—N2	1.482 (3)	C29—H29	0.95
C4—C5	1.506 (3)	C30—C31	1.383 (3)
C4—H4A	0.99	C30—H30	0.95
C4—H4B	0.99	C31—C32	1.387 (3)
C5—N4	1.343 (3)	C31—H31	0.95
C5—C6	1.385 (3)	C32—C33	1.389 (3)
C6—C7	1.386 (3)	C32—H32	0.95
C6—H6	0.95	C33—H33	0.95
C7—C8	1.379 (3)	C34—C35	1.401 (3)
C7—H7	0.95	C34—C39	1.404 (3)
C8—C9	1.397 (3)	C34—B1	1.651 (3)
C8—H8	0.95	C35—C36	1.398 (3)
C9—N4	1.345 (3)	C35—H35	0.95
C9—C10	1.499 (3)	C36—C37	1.384 (3)
C10—H10A	0.98	C36—H36	0.95
C10—H10B	0.98	C37—C38	1.384 (3)
C10—H10C	0.98	C37—H37	0.95
C11—N2	1.489 (3)	C38—C39	1.395 (3)
C11—C12	1.507 (3)	C38—H38	0.95
C11—H11A	0.99	C39—H39	0.95
C11—H11B	0.99	C40—C45	1.400 (3)
C12—N1	1.347 (3)	C40—C41	1.415 (3)
C12—C13	1.383 (3)	C40—B1	1.653 (3)
C13—C14	1.387 (3)	C41—C42	1.386 (3)
C13—H13	0.95	C41—H41	0.95
C14—C15	1.377 (3)	C42—C43	1.391 (3)
C14—H14	0.95	C42—H42	0.95
C15—C16	1.397 (3)	C43—C44	1.386 (3)
C15—H15	0.95	C43—H43	0.95
C16—N1	1.351 (3)	C44—C45	1.395 (3)
C16—C17	1.496 (3)	C44—H44	0.95
C17—H17A	0.98	C45—H45	0.95
C17—H17B	0.98	N2—Mn1	2.1828 (18)
C17—H17C	0.98	N4—Mn1	2.3522 (18)
C18—C19	1.381 (6)	N1—Mn1	2.3251 (18)
C18—H18A	0.98	O1—Mn1	1.8325 (15)
C18—H18B	0.98	O1—Mn1^i^	1.8349 (15)
C18—H18C	0.98	Mn1—Mn1^i^	2.6825 (7)
			
C1—N3—Mn1	119.9 (3)	C20—C21—H21C	109.5
C1—N3—H1A	107.3	H21A—C21—H21C	109.5
Mn1—N3—H1A	107.3	H21B—C21—H21C	109.5
C1—N3—H1B	107.3	C27—C22—C23	115.0 (2)
Mn1—N3—H1B	107.3	C27—C22—B1	123.0 (2)
H1A—N3—H1B	106.9	C23—C22—B1	121.9 (2)
N3—C1—C2	112.4 (4)	C24—C23—C22	122.9 (2)
N3—C1—H1C	109.1	C24—C23—H23	118.5
C2—C1—H1C	109.1	C22—C23—H23	118.5
N3—C1—H1D	109.1	C25—C24—C23	120.0 (2)
C2—C1—H1D	109.1	C25—C24—H24	120
H1C—C1—H1D	107.9	C23—C24—H24	120
C3—C2—C1	114.2 (4)	C24—C25—C26	119.1 (2)
C3—C2—H2A	108.7	C24—C25—H25	120.5
C1—C2—H2A	108.7	C26—C25—H25	120.5
C3—C2—H2B	108.7	C25—C26—C27	119.9 (2)
C1—C2—H2B	108.7	C25—C26—H26	120
H2A—C2—H2B	107.6	C27—C26—H26	120
C2—C3—N2	116.7 (6)	C26—C27—C22	123.0 (2)
C2—C3—H3A	108.1	C26—C27—H27	118.5
N2—C3—H3A	108.1	C22—C27—H27	118.5
C2—C3—H3B	108.1	C29—C28—C33	115.0 (2)
N2—C3—H3B	108.1	C29—C28—B1	124.39 (19)
H3A—C3—H3B	107.3	C33—C28—B1	120.48 (19)
C1B—N3B—Mn1	126.7 (19)	C28—C29—C30	122.8 (2)
C1B—N3B—H1B1	105.6	C28—C29—H29	118.6
Mn1—N3B—H1B1	105.6	C30—C29—H29	118.6
C1B—N3B—H1B2	105.6	C31—C30—C29	120.2 (2)
Mn1—N3B—H1B2	105.6	C31—C30—H30	119.9
H1B1—N3B—H1B2	106.1	C29—C30—H30	119.9
C2B—C1B—N3B	109.8 (15)	C30—C31—C32	118.9 (2)
C2B—C1B—H1B3	109.7	C30—C31—H31	120.6
N3B—C1B—H1B3	109.7	C32—C31—H31	120.6
C2B—C1B—H1B4	109.7	C31—C32—C33	120.2 (2)
N3B—C1B—H1B4	109.7	C31—C32—H32	119.9
H1B3—C1B—H1B4	108.2	C33—C32—H32	119.9
C1B—C2B—C3B	113.8 (17)	C32—C33—C28	122.9 (2)
C1B—C2B—H2B1	108.8	C32—C33—H33	118.6
C3B—C2B—H2B1	108.8	C28—C33—H33	118.6
C1B—C2B—H2B2	108.8	C35—C34—C39	115.2 (2)
C3B—C2B—H2B2	108.8	C35—C34—B1	123.53 (19)
H2B1—C2B—H2B2	107.7	C39—C34—B1	121.25 (19)
N2—C3B—C2B	115 (3)	C36—C35—C34	122.9 (2)
N2—C3B—H3B1	108.4	C36—C35—H35	118.6
C2B—C3B—H3B1	108.4	C34—C35—H35	118.6
N2—C3B—H3B2	108.4	C37—C36—C35	120.1 (2)
C2B—C3B—H3B2	108.4	C37—C36—H36	120
H3B1—C3B—H3B2	107.5	C35—C36—H36	120
N2—C4—C5	112.64 (17)	C38—C37—C36	118.9 (2)
N2—C4—H4A	109.1	C38—C37—H37	120.6
C5—C4—H4A	109.1	C36—C37—H37	120.6
N2—C4—H4B	109.1	C37—C38—C39	120.4 (2)
C5—C4—H4B	109.1	C37—C38—H38	119.8
H4A—C4—H4B	107.8	C39—C38—H38	119.8
N4—C5—C6	122.8 (2)	C38—C39—C34	122.5 (2)
N4—C5—C4	117.05 (18)	C38—C39—H39	118.7
C6—C5—C4	119.99 (19)	C34—C39—H39	118.7
C5—C6—C7	118.2 (2)	C45—C40—C41	114.69 (19)
C5—C6—H6	120.9	C45—C40—B1	124.37 (18)
C7—C6—H6	120.9	C41—C40—B1	120.93 (18)
C8—C7—C6	119.6 (2)	C42—C41—C40	123.2 (2)
C8—C7—H7	120.2	C42—C41—H41	118.4
C6—C7—H7	120.2	C40—C41—H41	118.4
C7—C8—C9	119.2 (2)	C41—C42—C43	120.1 (2)
C7—C8—H8	120.4	C41—C42—H42	119.9
C9—C8—H8	120.4	C43—C42—H42	119.9
N4—C9—C8	121.3 (2)	C44—C43—C42	118.5 (2)
N4—C9—C10	118.12 (19)	C44—C43—H43	120.7
C8—C9—C10	120.59 (19)	C42—C43—H43	120.7
C9—C10—H10A	109.5	C43—C44—C45	120.7 (2)
C9—C10—H10B	109.5	C43—C44—H44	119.7
H10A—C10—H10B	109.5	C45—C44—H44	119.7
C9—C10—H10C	109.5	C44—C45—C40	122.8 (2)
H10A—C10—H10C	109.5	C44—C45—H45	118.6
H10B—C10—H10C	109.5	C40—C45—H45	118.6
N2—C11—C12	110.51 (17)	C3B—N2—C4	103.8 (15)
N2—C11—H11A	109.5	C3B—N2—C11	110.0 (17)
C12—C11—H11A	109.5	C4—N2—C11	108.97 (16)
N2—C11—H11B	109.5	C4—N2—C3	111.9 (4)
C12—C11—H11B	109.5	C11—N2—C3	107.8 (4)
H11A—C11—H11B	108.1	C3B—N2—Mn1	123.8 (11)
N1—C12—C13	122.8 (2)	C4—N2—Mn1	104.85 (12)
N1—C12—C11	115.39 (18)	C11—N2—Mn1	104.74 (12)
C13—C12—C11	121.7 (2)	C3—N2—Mn1	118.1 (3)
C12—C13—C14	118.1 (2)	C5—N4—C9	119.01 (18)
C12—C13—H13	121	C5—N4—Mn1	109.15 (13)
C14—C13—H13	121	C9—N4—Mn1	131.23 (14)
C15—C14—C13	119.6 (2)	C12—N1—C16	119.17 (19)
C15—C14—H14	120.2	C12—N1—Mn1	110.88 (14)
C13—C14—H14	120.2	C16—N1—Mn1	129.95 (15)
C14—C15—C16	119.8 (2)	Mn1—O1—Mn1^i^	94.02 (7)
C14—C15—H15	120.1	C28—B1—C22	109.51 (17)
C16—C15—H15	120.1	C28—B1—C34	112.06 (17)
N1—C16—C15	120.5 (2)	C22—B1—C34	108.04 (17)
N1—C16—C17	117.82 (19)	C28—B1—C40	108.53 (17)
C15—C16—C17	121.7 (2)	C22—B1—C40	110.73 (17)
C16—C17—H17A	109.5	C34—B1—C40	107.96 (17)
C16—C17—H17B	109.5	O1—Mn1—O1^i^	85.98 (7)
H17A—C17—H17B	109.5	O1—Mn1—N3B	94.3 (6)
C16—C17—H17C	109.5	O1^i^—Mn1—N3B	177.0 (11)
H17A—C17—H17C	109.5	O1—Mn1—N3	89.11 (13)
H17B—C17—H17C	109.5	O1^i^—Mn1—N3	175.08 (13)
C19—C18—H18A	109.5	O1—Mn1—N2	174.90 (7)
C19—C18—H18B	109.5	O1^i^—Mn1—N2	89.04 (7)
H18A—C18—H18B	109.5	N3B—Mn1—N2	90.7 (6)
C19—C18—H18C	109.5	N3—Mn1—N2	95.86 (13)
H18A—C18—H18C	109.5	O1—Mn1—N1	106.39 (7)
H18B—C18—H18C	109.5	O1^i^—Mn1—N1	94.30 (6)
C18—C19—O2	108.7 (4)	N3B—Mn1—N1	82.8 (10)
C18—C19—H19A	109.9	N3—Mn1—N1	87.4 (2)
O2—C19—H19A	109.9	N2—Mn1—N1	75.08 (6)
C18—C19—H19B	109.9	O1—Mn1—N4	103.70 (6)
O2—C19—H19B	109.9	O1^i^—Mn1—N4	93.77 (6)
H19A—C19—H19B	108.3	N3B—Mn1—N4	89.1 (11)
C20—O2—C19	112.4 (4)	N3—Mn1—N4	87.0 (2)
O2—C20—C21	106.6 (3)	N2—Mn1—N4	75.49 (6)
O2—C20—H20A	110.4	N1—Mn1—N4	149.29 (6)
C21—C20—H20A	110.4	O1—Mn1—Mn1^i^	43.03 (5)
O2—C20—H20B	110.4	O1^i^—Mn1—Mn1^i^	42.96 (5)
C21—C20—H20B	110.4	N3B—Mn1—Mn1^i^	137.2 (6)
H20A—C20—H20B	108.6	N3—Mn1—Mn1^i^	132.13 (12)
C20—C21—H21A	109.5	N2—Mn1—Mn1^i^	131.99 (5)
C20—C21—H21B	109.5	N1—Mn1—Mn1^i^	104.13 (5)
H21A—C21—H21B	109.5	N4—Mn1—Mn1^i^	101.93 (5)

Symmetry code: (i) −*x*+1, −*y*+2, −*z*+1.
